# Identification of the Suprascapular Nerve with Minimal Volume of Contrast Medium: A Cadaveric Case Study

**DOI:** 10.37825/2239-9747.1057

**Published:** 2024-08-01

**Authors:** Luca Miceli, Giulia Bongiorno, Andrea Tomasi, Riccardo Lo Cascio, Giacomo Passoni, Francesca Dal Mas, Helena Biancuzzi, Rym Bednarova, Maria Cristina Melia, Marco Cascella, Alessandro Vittori

**Affiliations:** a Department of Pain Medicine, IRCCS C.R.O. National Cancer Institute of Aviano, Pordenone, Aviano, Italy; b “Friuli Riabilitazione” Rehabilitation Center, Roveredo in Piano, Pordenone, Italy; c Department of Orthopedic Surgery, Papa Giovanni XXIII Hospital, Monastier di Treviso (city of Treviso), Italy; d Department of Orthopedic Surgery “Latteri Clinic-Tigano Group”, City of Palermo, Italy; e “The Lab” Rehabilitation Center, Udine, Italy; f Department of Management, Ca, Foscari University of Venice, Venice, Italy; g Department of Economics, Ca, Foscari University of Venice, Venice, Italy; h Pain Medicine, Hospital of Latisana, Udine, Latisana, Italy; i University of Salerno, School of Medicine, Salerno, Italy; j Department of Anesthesia and Critical Care, ARCO Roma, Ospedale Pediatrico Bambino Gesù IRCCS, Rome, Italy

**Keywords:** Locoregional anesthesia, Cadaver, Pain, Opioid, Local anesthetic, Nerve

Painful shoulder pathology is particularly widespread and disabling in the population. This context requires an integrated approach between the orthopedist, the pain therapist, and the physiotherapist [1]. In particular, adequate pain control can be a support to the patient awaiting surgery, both in the rehabilitation process after surgery and in cases where surgery is not indicated (or health conditions of the patient contraindicate its execution).

For several years, methods of neuromodulation of the suprascapular nerve have been developed, in addition to pharmacological therapies. The goal is to guarantee adequate pain control by limiting the painful afferents coming from the shoulder district. Pulsed radiofrequency can guarantee good pain control without repercussions on the motor function of the treated nerve and is also used in shoulder pathologies [2]. In this district, pulsed radio-frequency has a very variable success rate, ranging from 50 to 90%; before proposing this neuromodulation method to the patient, clinicians often perform a block test with local anesthetic of the suprascapular nerve [3]. This operation has the aim of calibrating the treatment on a correct identification of the patient’s pain generator and therefore obtaining diagnostic confirmation [4]. The minimum volumes of pharmacological mixture suggested in the literature for blockade of the suprascapular nerve are generally 5–10 ml [4]. This quantity has also been confirmed by studies on cadavers, always with volumes around 10 ml, to allow adequate imbibition of the suprascapular nerve [5].

**Fig. 1:**
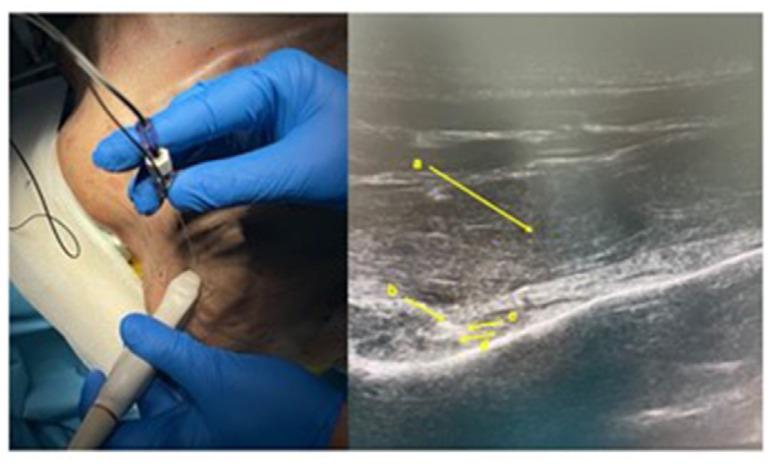
Left suprascapular nerve block US guided. A: needle; B: needle tip; C: suprascapular nerve; D: suprascapular artery.

The working hypothesis proposed in this single cadaveric case study was to reduce the volume used during the execution of the test to obtain greater accuracy in the identification of the suprascapular nerve and therefore ultimately power, once proposed on a large scale, reduce the rate of the ineffectiveness of the pulsed radiofrequency of this nerve, which could be due in part to an incorrect diagnostic classification with incorrect identification of the patient’s pain generator during the testing phase with a local anesthetic. This hypothesis derives from the work of Sinha et al., of 2019 who successfully used a test with a very low volume of local anesthetic, 2 ml, which is why it was decided to use the same volume on the cadaveric preparation [4]. On a cadaveric specimen, an infiltration of the left suprascapular nerve was performed under ultrasound guidance with a 12 Hz linear probe with a 100 mm long needle, using a small volume of colored contrast, 2 ml of methylene blue (Fig. 1).

**Fig. 2:**
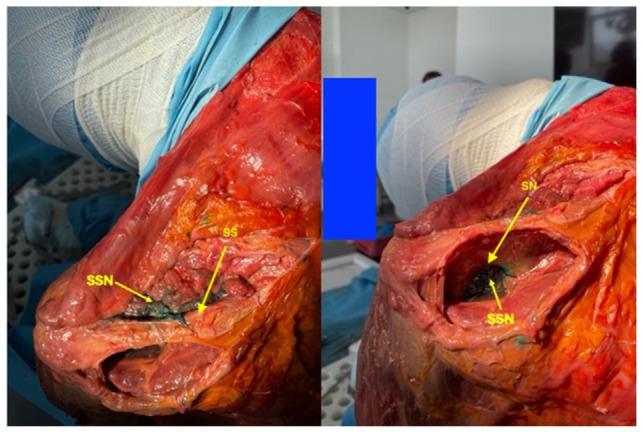
Imbibition of the suprascapular nerve, in its supraspinatus and infraspinatus component, with 2 ml of methylene blue. SSN: suprascapular nerve; SN: scapular notch; SS: scapular spine.

The anatomical specimen was in a sitting position. The needle was inserted at an angle of approximately 45° to the skin, and the nerve was identified at a depth of 3.5 cm from the skin. Once the dissection of the anatomical preparation was then carried out, it was seen how the contrast had spread to bathe the suprascapular nerve for over 4 cm of its course, both in its component that runs through the supraspinatus and infraspinatus fossa (Fig. 2). The clear visibility of the contrast medium above and below the spine of the left scapula demonstrates that the fluid has traveled through the scapular notch, where the suprascapular nerve runs. The inspiration offered by this single case, if confirmed by larger series, is that even “in vivo” a volume of 2 ml may be sufficient to perform a block of the suprascapular nerve with local anesthetic, reducing the failure rate of any subsequent pulsed radiofrequency due to excessive imbibition of the tissues surrounding the nerve during the test with local anesthetic if the usual dosages equal to or greater than 5 ml are used. These dosages could, in fact, lead to an incorrect interpretation of a positive test of reduction of the patient’s pain, due to a local effect of imbibition of the shoulder tissues by local anesthetic rather than to a correct peripheral nerve block. This precision in nerve localization is of great importance since the active part of the tip of the pulsed radiofrequency needles is normally 5 mm, i.e. capable of generating a virtual sphere of neuromodulation of approximately 10 mm (twice the radius of 5 mm) and therefore being able to wet at least 20 mm of nerve during the test with local anesthetic could be sufficient for a positive prognostic predictive effect of the test.
